# About Platinum

**Published:** 1890-06

**Authors:** 


### About Platinum.

Charles Wood, an assayer, in 1741 found in Jamaica some platina which had been brought from Carthagena and which he forwarded to London for inspection as a curiosity.

The first to mention platina by its present name, however, which means "little silver," was Don Antonio Ulloa, a Spanish mathematician, who, in 1735, accompanied the French academicians who were sent to Peru by their sovereign to measure a degree of the meridian in order to determine the figure of the earth.

After his return he published at Madrid, in 1748, a history of his voyage, and mentioned the abandonment of the gold mines in the territory of Choco on account of the presence of platina, which, being too hard to easily break or calcine, the gold could not be extracted without much expense and great difficulty.

It is reported in the *Chemical Annals* for July, 1792, that the miners of Choco, discovering platina was a metal, began to use it in adulterating gold, in consequence of which the Court of Spain, fearing disastrous results therefrom, attempted not only to prevent its export, but to conceal the discovery of the metal from the world.

To effect this all gold brought from Choco to be coined at the two mints of Santa Fe was carefully inspected, and all platina separated and given to the king's specially appointed officers, and when a sufficient quantity had accumulated, it was taken to the river Bogota, about two leagues from Santa Fe, or to the river Cauca, about one league from Papayan, and in the presence of witnesses thrown into the river.

From the great specific gravity of this metal, it being the heaviest known, together with its malleability and ductility, and the fact of its great resistance to the action of acids, alkalies and sulphurs, it has become known as the "metal of the chemists."

Some of the most important discoveries of modern chemistry would have been impossible without the aid of platina. It is so soft it may be readily cut with the scissors, and when formed into a mirror, reflects but one image.

Platina has been found in various parts of the world—Peru,

New Granada, Brazil, St. Domingo, and in the gold washings of California, Australia and Borneo—but the principal source of supply is in the Ural Mountains of Russia and the auriferous sand of Kuschwa, in the Auralian Mountains of Siberia.

Platina is rarely found in pieces larger than a few grains in weight.

The chief uses of platina are for the various apparatus used in chemical laboratories, such as crucibles (first crucible was produced in 1784), spoons, blow-pipe points, tongs, forceps, and boilers or stills for concentrating sulphuric acid. A still of this kind, valued at 95,000 francs, was exhibited at Vienna in 1878, capacity 20,000 pounds of sulphuric acid daily.

An ingot valued at £4,000 was exhibited at the London Exhibition in 1862.

On account of the high degree of heat requisite to fuse or melt platina—melting point 1,460° to 1,480°—it is the only metal used for making the pins of porcelain teeth, and on account of its value and lack of any known substitute, has become the greatest item of expense in their manufacture.

It is also utilized for making fine jewellery, and a great and growing demand has been but recently created by the development of electricity.

The Russian Government began coining platina for general circulation in 1826 and continued until 1845, when by an imperial ukase the coinage was discontinued and the £500,000 issued called in because of the great fluctuation in the price of the metal.

The average production of platina metal from 1828 to 1845 amounted to 2,623.8 kilos or 5,784.48 lbs. per annum ; from 1875 to 1884 inclusive, the average yield of the Russian mines was 3,483.3 lbs. per annum, showing a decrease since 1882, the maximum year of 45 per cent. in the yield. The Russian mines yield 80 per cent. of the total product of the world.

The price of platina, which has always ruled very high in consequence of the continually increasing demand, the limited source of supply, without any new discoveries of moment sufficient to relieve the market, is constantly advancing, so rapidly indeed as to cause serious apprehension for the future.

Those industries whose manufacturers depend largely on platina as their chief element of cost (and with no known substitute in sight), such as crucibles, porcelain teeth, electrical and chemical apparatus, etc., are suffering more or less seriously from this increase in price, and for self-protection it would seem will be obliged to advance prices proportionately.—*Electrical Review.*

